# From activism to secrecy: Contemporary experiences of living with HIV in London in people diagnosed from 1986 to 2014

**DOI:** 10.1111/hex.12816

**Published:** 2018-08-30

**Authors:** Tanvi Rai, Jane Bruton, Sophie Day, Helen Ward

**Affiliations:** ^1^ School of Public Health Imperial College London London UK

**Keywords:** chronic disease, continuity of patient care, HIV clinic, HIV infection, normalization, privacy, public health, self‐care, social stigma, social support

## Abstract

**Background:**

Successes in biomedicine have transformed HIV from a debilitating and frequently fatal infection to a chronic, manageable condition.

**Objective:**

To explore how the contemporary metanarrative of HIV as a chronic condition is understood by patients and how it varies depending on when they were diagnosed.

**Design:**

Qualitative interviews with 52 people living with HIV who were diagnosed during different phases in the history of the epidemic.

**Setting and participants:**

Participants were recruited from two HIV clinics in London to include four “HIV generations”: generation 1 were those who had been diagnosed pre‐1997 (pre‐ART), generation 2 from 1997 to 2005 (complex ART), generation 3 from 2006 to 2012 (simpler ART) and generation 4 diagnosed in the year before the study (2013‐2014).

**Results:**

Participants in all HIV generations took their medication as prescribed, attended clinic appointments and were well informed about their immunological biomarkers. While the pre‐treatment generation had been engaged in community endeavours such as activism, public education and use of support groups, those more recently diagnosed had little experience of collective activities and their HIV was essentially a private matter, separate from their social identity. These strategies worked for some; however, those experiencing clinical or social problems related to HIV or wider issues often relied exclusively on their HIV clinic for wider support.

**Conclusion:**

The loss of public conversation around HIV, the imperative for patients to take on greater individual responsibility for HIV management and the streamlining of HIV services alongside reductions in ancillary support services may expose some people to suboptimal health outcomes.

## 1 INTRODUCTION

Antiretroviral therapy (ART) development and the results from clinical trials have transformed the management of HIV and the organization of clinical services. It has become a showpiece for successful translational medicine. In the UK, newly diagnosed patients are encouraged to start ART immediately, and once stable on treatment are seen once or twice a year at a specialist HIV clinic. For all other medical support, patients are advised to visit their general practitioner (GP), who is expected to be aware of, and engaged with, their patients' HIV status and care.^1^, ^2^

Alongside scientific and clinical developments, the social relationships established in response to HIV infection have also changed. In the 1980s and early 1990s in the global north, HIV was the “killer” infection that brought people together into communities under siege.^3^ Within the groups most affected (including gay men and African women), HIV served to mobilize and unite those facing the catastrophe together, albeit in different ways. There were few medical solutions, and therefore, social and psychological support was crucial. Social activism invoked principles of equal rights and global citizenship to draw public attention and institutional commitment towards universal access to care (and treatment when it came) for HIV patients.^4^ Even individuals who did not ascribe to a common identity or community in relation to HIV (e.g. white heterosexuals, black men and people who inject drugs) gained access to a collective of doctors, nurses, health advisors, care workers, housing officers and volunteers who devoted their professional and often personal lives to providing palliative, psychological and practical support, as well as joining movements for social change.^5^ A major part of HIV management in the time before effective ART also involved patients talking and sharing their experiences of the challenges of living with HIV^6^ and, in doing so, contributing to a collective consciousness about HIV.

Thanks to the success of ARTs, HIV has now been transformed from “death sentence to life sentence” (pp. 665‐679).^7^ The simplification and effectiveness of treatment mean that the infection is not necessarily life‐shortening,^8^ treatments have fewer side‐effects, and, while on effective treatment, people with HIV do not transmit the virus to others.^9^, ^10^ These optimistic developments in HIV science beckon us to renounce the historical crisis response and replace it with efforts towards normalization, a process encompassing a shift away from historical AIDS exceptionalism and the reframing of HIV as chronic disease “like any other”.^11^ In Western Europe, access to ART is universal, with patients expected to act as partners, sharing responsibility to keep their viral load undetectable by early initiation and careful adherence to ART. It is therefore anticipated that they will become self‐regulating, healthy citizens who are “biomedicine's partners in a normalized enterprise of survival, and as far as possible, healthy living”^12^ (p. 8).

The streamlining of HIV care in the UK may also have an unintended effect of undermining or even silencing historical and continuing discussion on the social and psychological effects of HIV infection, its unequal distribution in terms of structural disadvantages and links with other health issues.^13^, ^14^ As HIV remains a socially pathologized and stigmatizing condition despite the great leaps in biomedicine,^11^ it is likely that barriers will continue to hinder prevention, treatment, care and support.^15^

In this study, we explore how the contemporary metanarrative of HIV as a normalized, chronic disease is understood and experienced by people living with HIV and ask whether it varies for people who were diagnosed at different points of the epidemic. People's experience of HIV differs by when they were diagnosed and also by their diverse backgrounds, treatment histories, overall health, comorbidities and social support. By including patients diagnosed at different points in the path towards effective HIV treatment, we aim to chronicle patient stories including how they have adapted to biomedical developments.

## 2 METHODS

We conducted a qualitative study of people attending two large London HIV clinics. Prior to starting the study, we conducted a focus group discussion in which six patients were invited (four attended) to discuss the topic guide, study design and approach and other issues that they felt were relevant to people living with HIV. Ethical approval was obtained from NRES (reference number 14/WM/0147) in May 2014, and research governance approval was obtained from the local sites.

### 2.1 Sample and study design

Detailed recruitment and sampling methods are published elsewhere.^16^ Briefly, we recruited 52 study participants from September 2014 to April 2015. To reflect the evolution of ART, we had identified four “HIV generations” on the basis of time of diagnosis: generation 1 were those who had been diagnosed pre‐1997 (pre‐ART), generation 2 from 1997 to 2005 (complex ART), generation 3 from 2006 to 2012 (simpler ART) and generation 4 diagnosed in the year before the study (2013‐2014). Within each generation, we aimed to include people with a range of characteristics (such as age, sexual orientation, gender and ethnicity). Although generations are not directly comparable—some have longer histories compared to others—it provided an opportunity to explore changes over time in terms of the physical, social and health service experience of living with HIV. Participants were recruited opportunistically by researchers attending clinical services and through fliers and digital screens advertising the study in clinical areas. Recruitment was periodically checked against the recruitment matrix whose primary function was to ensure similar numbers from the four HIV generations and diversity within each generation in terms of the above‐mentioned demographics. There were no exclusion criteria, and under‐represented strata/groups were targeted using clinic lists.

Study participants were provided with information about the study and gave written consent. Interviews took place in private rooms in or near the clinics or at the participant's home; they were recorded and transcribed and lasted between 60 and 90 minutes. Interviews were semi‐structured and carried out by four researchers (TR, JB and two others, see Acknowledgements) with open‐ended questions within a topic guide designed to elicit study participants' experiences during their HIV “journey” from the time before diagnosis until the present. We asked them about their health, employment status, relationships and social networks; their relationship with clinicians and decision making regarding HIV‐related matters; and about the role HIV played in their lives. We also asked for their views on taking ARTs (usually just one or two pills a day) and the reduced frequency of clinic appointments (among those immunologically stable). All audio recordings, field notes and transcription files were saved in an encrypted secure location on the university server, separate from consent forms and other identifying information.

### 2.2 Data analysis

We analysed interview transcripts through a combination of framework and thematic analysis.^17^, ^18^ Open coding procedures carried out independently by TR and JB led to the identification of an initial set of codes that were loosely structured around the general areas of interest identified in advance and additional emergent themes. Using an iterative process of multiple readings, coding, analyses and discussion, these themes were then modified, expanded or amalgamated to create a final coding frame which was then discussed with HW and SD and further analysed in relation to the existing literature. The final framework was then applied across the data set, leading to more in‐depth analysis where we synthesized coded data and explored relationships across themes and how they applied across different participants in the study sample. Data were managed using the software NVivo v9, (QSR international, Melbourne, Australia).

## 3 RESULTS

We recruited and interviewed 52 people, 25 at one clinic and 27 at the other.^16^ Our sample comprised of 37 men who have sex with men (MSM), 4 heterosexual men and 11 women, one of whom was infected through injecting drug use, the rest through sex with men. There were 11 in generation 1, 14 generation 2, 17 generation 3 and 10 generation 4. The generation samples differed somewhat by gender and acquisition: women were concentrated in generations 1 and 2 (6 and 4, respectively) and MSM in generations 3 and 4 (16 and 8, respectively). Recruiting women from later generations proved challenging and was indicative of the declining proportion of women in clinic listings of patients diagnosed since 2006.

All study participants, regardless of when they had been diagnosed, recognized HIV as a manageable, long‐term condition. Recently diagnosed participants had only experienced the care currently offered in HIV services, whereas those from earlier HIV generations had observed changes in services in the form of fewer appointments, seeing a nurse instead of a physician for some consultations, increased emphasis on self‐management and a greater involvement of GPs in managing their overall health.

### 3.1 Strategies for coping—then and now, together and alone

The experiences of those diagnosed in the pre‐ and early ART era, that is generations 1 and 2 of our sample, had been very different in the past, and they described a range of strategies which have almost disappeared in later generations. Participants from generation 1 had lived through many difficulties and were still haunted by memories of friends and lovers who had died and waiting rooms and wards full of very sick people. Their treatment histories varied: two of the 11 had started treatment with early monotherapy in the 1980s, while the rest delayed because of feared side‐effects. During this period, another two consciously disengaged from care, only reconnecting when their health deteriorated. Even after 1997, taking early ART meant several years of quite debilitating side‐effects and multiple changes of complex regimens until the arrival of better‐tolerated medications.

Coping with HIV in those early days was in part through involvement in HIV activism, joining the collective response in a range of ways. Nine people described becoming involved as campaigners, volunteers, peer support workers and activists in groups such as Terrence Higgins Trust and Positively Women/UK. They supported research as participants, advisors and lobbyists. In addition to any political impact, individuals like Rory (below, all names are pseudonyms) recalled how this helped them live with HIV:

I'd already joined Terrence Higgins Trust [THT] as a volunteer, so we were having a lot of helpful doctors, who, on top of their full‐time jobs, were giving up their time to come and give their time freely to the THT to educate people who wanted to help in the best way possible with the THT helpline and looking after people who were HIV. By going to a lot of the various meetings, workshops and things like that, I think I learnt more, probably, than the average person would have done at that time. (Rory, Gen1, MSM)

In contrast, participants from post‐ART generations appeared to have consciously rejected this approach, suggesting that HIV should claim only a small part of their social identity. This was particularly apparent in the next cohort, generation 2, where eight (of 14) participants foregrounded their HIV experience by describing the kind of person they did not want to be. In particular, they expressed disapproval of those they saw as “career HIV people”; instead, they praised the self‐reliance and HIV anonymity made possible by ART and distanced themselves from a public HIV identity associated with social activism. Andy, a gay man in his forties from generation 2, expressed his disdain for people who liked to “live their lives surrounded by gayness and HIV.” Likewise, Sheila, an African woman in her 50s, said that a HIV‐positive status was no excuse for becoming a “baby of the government,” citing her own example as someone who worked full‐time and was raising two children. Peter, in his 50s and also working full‐time, recalled patients from a support group he had attended after his HIV diagnosis as being “moany” and believed they should go and get jobs.

Several from generation 1 continue their activism: two women work as mentors for an HIV organization, another established a new support group outside London, and one woman provides HIV education in schools. Similarly, one man has established a series of sports initiatives in Africa to promote awareness of HIV, another is involved in health policy, and another does interviews on television and radio discussing life with HIV.

The importance of community and self‐help as a way of coping, and helping others, was far less prominent in later generations, who preferred to keep their HIV within the realm of the clinic. They welcomed the streamlining of treatment and care with fewer, shorter clinic appointments which meant they had to “think about it less.” They took their medicines and attended clinic appointments, ate well and exercised.

### 3.2 Embracing the numbers and becoming self‐reliant

Once past the initial stages of their diagnosis and care, which almost universally caused reactions of shock, updates on their viral loads and CD4 counts at clinic visits were felt to be reassuring and gave participants a sense of well‐being. For example, Adam got a thrill every time his monitoring tests revealed an undetectable viral load:

Going back to the original diagnosis where I just thought I wouldn't even live for another year, to think that I have come all this way and …I am just having to take like a handful of pills every day, and that is going to keep me alive until hopefully as long as anyone else. I just think when I hear that undetectable viral load that for me is like, you have got a life. Do you know what I mean? (Adam, Gen2, heterosexual man)

When talking about the present, three quarters of the sample, across all generations, described how they felt a sense of control over the condition by shrinking HIV into a tidy set of biological measurements. For example, Phil, a gay man in his late 30s from generation 3, who initially found the medicalized language surrounding HIV off‐putting and stressful later found himself hooked on the numbers, becoming quite obsessed and competitive about maintaining his CD4 count at >1000 (“God, I love those numbers!”). A few also participated enthusiastically in trials and enjoyed the approval from clinicians when they demonstrated high levels of knowledge. Another participant said: “You have to manage the disease, the disease can't manage you.”

However, this rhetoric of self‐reliance and empowerment existed alongside accounts suggesting a continuing sense of vulnerability. For instance, Phil, mentioned above, was doing very well on his biomarkers but had continued to need significant psychological support from his HIV clinic.

I had an episode recently. I came into the clinic and I saw [name of doctor]. (…) I've known him long enough that he can be… He's a bit human. Everybody is human here, and they have a bit of a chat and say, ‘How are you?’ (…) and I said something like, ‘Do you mean how am I or how am I, clinically?’ (…) I said two words and then I burst into tears for 40 minutes. I had split up with my partner, a friend had just died of cancer having given birth to an HIV baby, and I was stressed at work. I fell apart, technically in the right place at the right time because, the very next day, there was a fast‐track psychological service that was being launched. (Phil, Gen3, MSM)

At the time of interview, Phil was suffering side‐effects from medication and had lost his job. His frequent trips to the clinic for medical reasons had given him an opportunity to discuss and receive support for these other issues as well.

### 3.3 Living normally—keep it to yourself

Along with becoming familiar with their HIV‐related numbers, interviewees generally aimed to restrict HIV to the clinic and lived “normally” by concealing their status. We did not set out to explore disclosure, but participants across all generations described problems they had faced following HIV diagnosis and often felt more comfortable keeping anything related to HIV private. Asked about her advice for newly diagnosed people, Sheila (mentioned above) said:

Some people get depressed, okay, some people get even mentally affected by that and they go bananas, they go telling people, ‘Oh, the doctors have just diagnosed me with HIV.’ You are bringing stigmatisation and self‐pity on yourself! (…) Keep it to yourself…(…) When you keep it to yourself as much as you can and then you carry on with your life. (Sheila, Gen2, female)

Aside from sharing their diagnosis with existing partners, one or two family members or HIV‐positive friends, participants in the three later generations were isolated in their HIV experiences except when they visited the clinic. Five (of 31) participants across generations 2 and 3 had briefly attended HIV‐positive support groups soon after diagnosis, while none (of 10) from generation 4 had at the time of interview.

Some aspects of concealment regarding HIV status appeared specific to gay men. Seven gay men from across the three later generations commented specifically on the parallels between keeping HIV secret and managing homophobia. Secrecy constituted a strategy for avoiding stigma and discrimination. Having learned, often through unpleasant experiences, the value of maintaining control over who to tell about their sexual orientation, they applied the same approach to their HIV status. For example, Gareth described the stigma of HIV being like a “second coming out” and attributed the loss of his senior job to institutional homophobia. With HIV, he decided early on not to share his status with anybody, saying he was “…kind of in an HIV positive vacuum…” Gareth did not disclose his HIV status to sexual partners and felt justified by his undetectable viral load—a consequence of adherence to ART treatment—that there was no real need to disclose.

I do lead, broadly speaking, certainly from anybody else's perspective outside looking in, no different an existence to when I was HIV negative and my status was different. (Gareth, Gen3, MSM)

In contrast, the five gay men in generation 1 reported having been open about both their sexuality and their HIV status as part of their identity linked to activism.

Managing disclosure to partners was an on‐going concern for others too, across all generations. Anita, from generation 2, recently divorced with two young children, had delayed disclosing her status to her new boyfriend until she was certain the relationship would last. Likewise, Henry, in his early 30s from generation 4, described how he took a more structured and long‐term approach to dating girls since his diagnosis, partly to manage the risk of being judged and possibly rejected but also because “you can't un‐tell someone” especially if the relationship did not last:

…we've had to have this conversation, and now she knows that I'm HIV positive. You know, she knows intimate things about me, and now I've decided that actually we probably couldn't make this work. You know, it's that flip side as well. So I guess it makes dating a lot more serious. (Henry, Gen4, heterosexual man)

Overall three quarters of our study participants were in reasonable health and had found different strategies to live with their HIV. However, they expressed apprehension about the future in which near‐average life expectancy was accompanied by risks of comorbidities and the possibility of needing support from family and friends who may not be aware of their HIV.

### 3.4 Difficult times

The fears for the future were exacerbated among the remaining quarter of our cohort, who were not coping particularly well due to some combination of HIV‐related ill health, troubling comorbidities, mental health problems, domestic abuse or financial insecurity. For these people, who spanned the generations, the longer clinic appointments immediately following diagnosis had provided an opportunity to talk freely and privately with sympathetic and non‐judgemental professionals. The on‐going relationship with specialist staff had proved helpful in managing a range of health problems, including ART adherence. For example, Grace (generation 2) recalled how the combination of living with an abusive husband, working full‐time and caring for her young children pushed her into a depression and she stopped taking her ART medication. Her consultant recognized her distress, referred her for counselling and advised her on obtaining a better work‐life balance.

A more sustained emotional dependency on the clinic was apparent for half of this group. For example, Zach felt isolated from his family who did not know that he was gay. On becoming HIV positive, he missed clinic appointments, pulled away from his friends, changed jobs and went through a phase of intensive drug and alcohol use and multiple sexual partners. Acquiring hepatitis C finally induced him to attend regular appointments with his clinical nurse specialist (CNS), with whose help he gradually felt better:

The nurse I've got now is [], (…) In essence, that's probably the main reason I don't.(…) It's really bad, isn't it, that I'm thinking, ‘I better not do this because the nurse is going to give me the – ‘Really?! ‐’? (…) If he wasn't [available], you know what, I'd probably go off the rails a bit more. I'm being completely honest. It's a shame I'm not seeing him more often. (Zach, Gen3, MSM)

Similarly, Katie, recently diagnosed, was a single, young mother living on benefits who had recently left an abusive relationship. She spoke of her CNS as a confidante and friend, the like of whom she had never met before.

With [name of CNS], she's seen the tears, she's been the reassurance, she's gone through the tantrums, where I can't cope with it, any worries or concerns, even my day to day life, my family, she was there. She was like the person I was in a relationship with (…) So [she] was the back bone, if you want to put it ‐ for me, when it comes to this illness. I always say if it wasn't for her, I don't think I could ever have coped with it the way ‐ even though, by the sounds of it, I haven't really coped with it, but I would have gone to pieces, if I'm honest. (Katie, Gen4, female)

Rory, mentioned earlier as an activist from generation 1, was suffering from a number of health problems and chronic pain. He linked the changes to the clinic services, with fewer, shorter appointments to broader cuts in welfare and social support which all made it harder to manage his complex condition. In addition, being told that he no longer had AIDS but only HIV felt like a betrayal and a way of “shoving sick people back to work,” a reference to changes in disability benefits in the UK.

Well, because I'm not making a valid contribution and I know that I can't [challenge the service changes]. If I could, I would, although again, I think that because the government's attitude is ‘Everybody must be at work regardless,’ it's a very damaging thing. It also means that, even whereas before, years ago, you could volunteer to do things and that would be warmly welcomed and accepted, these days, it's more a case of ‘Well, if you can do that, you don't need to be on this benefit. You can get on the unemployment register and go back to work. (Rory, Gen1, MSM)

## 4 DISCUSSION

Our study of 52 people living with HIV has identified both similarities and differences between generations diagnosed at different points in the epidemic. We have identified a rupture in the way people in the pre‐ and post‐ART generations relate to their condition: people diagnosed pre‐ART described involvement and activism through political campaigns and support groups, while those in the following generation consciously rejected such activities that reminded them of their HIV‐positive identity, finding greater resilience through isolating themselves from such community processes. Those diagnosed during the era of effective and simple ART were not exposed to the AIDS activism of the 1980s and 1990s and had only experienced HIV as something private.

At the time of interview, the majority of our participants were virally suppressed, financially stable and healthy. They engaged with the HIV clinic and their treatment and gained confidence in self‐management through monitoring their biomarkers which indicated that their HIV was under control. At the same time, the post‐ART generations kept their seropositive status hidden from most other people, including family, friends and sometimes even sexual partners. These two strategies for living with HIV related to their recognition that HIV stigma remained a potential threat to their well‐being.

Resonating with a recent study also based in the UK,^19^ the confidence projected by the majority of study participants about their successful self‐management, confirmed by measurements that showed that the virus was under control, existed alongside lingering insecurities that threatened their carefully arranged lives. The difference between those who did and did not manage HIV “well” was not static but shifting whereby participants might experience periods of physical or emotional difficulty and uncertainty, followed by other periods when life became easier. A quarter of those we interviewed were in frequent contact with the clinic and other services for support with complications and comorbidities, some of whom appeared very dependent on their clinicians.

The determination of people living with HIV to embrace the metanarrative of HIV normalization is challenged by the ignorance of wider society about the radical transformations in HIV biomedicine,^19^, ^20^ making it difficult to be open about their HIV status. In doing so, it reduces “the ‘social problem’ of HIV to an individualized concern”^21^ (p. 509). One theme that unified many accounts of our study participants (aside from generation 1) was isolation; they had largely experienced HIV alone. Other writers have noted how the rebranding of HIV from its original form as “an exceedingly public illness”^22^ (p. 1066) into an individualized and private problem has entailed the loss of collective HIV activism and avenues for discussion about HIV, leaving people largely on their own to navigate the social, psychological and structural challenges that remain significant.^3^ In our study, most respondents after generation 1 had stayed away from support groups, concealed their status from their social networks and kept HIV‐related matters separate even from other parts of their *own* life. In demonstrating their comfortable grasp of biomedical measures of immune status and therapeutic developments, study participants were enacting what Thompson and Abel describe as the “domestication” of the disease.^23^ However, unlike many other chronic diseases, the need for secrecy was crucial, and the normalization of life with HIV rested on a strategy of concealment, a strategy that was reported more than 20 years ago, “…secrecy is the central way of managing everyday life for one's self and for others. It was necessary to keep the secret in order to live as normally as possible…” (p. 74).^24^

Goffman^25^ observed that stigma is experienced differently according to how easy it is to *conceal* the stigmatizing attribute. If the associated attribute is visible (gender, ethnicity), individuals bearing the attribute are *discredited;* however, when it can be concealed (HIV, mental illness), individuals are *discreditable*.^25^ Goffman's concept of *passing* describes the management of undisclosed discrediting information about the self. It allows people with a discreditable stigma to “pass” in public as though they were “normal.” While modern management of HIV is associated with reduced discrimination as people generally look well, evidence is accumulating about significant psychological costs associated with the effort to maintain privacy.^26^, ^27^ The prevalence of psychiatric and psychological problems is significantly higher in those with HIV compared to the general population.^28^ This process of hiding a condition largely precludes collective strategies and support and can lead to a vicious cycle: secrecy about HIV prevents positive collective action to change public perceptions of HIV and so, privately, those with HIV continue to suffer psychological morbidity, which in turn leads them to avoid public action. In addition to psychosocial issues, the intersection of HIV with other chronic health problems as people with HIV age has increased the need for social and sometimes disability services. However, concurrent structural changes in the provision of health and social care have reduced access to appropriate support.

### 4.1 Strengths and limitations

The strengths of this study are in the inclusion of a diverse sample of people with HIV diagnosed over 30 years. By focusing on generations, we have uncovered important changes in the ways that people with HIV manage their condition over time, both as individuals and collectively. Our research was based in London, and therefore, the findings may not be generalizable to other health‐care settings, especially outside the UK. The sample had a disproportionate number of women in the earlier generations compared to later generations; therefore, it is possible that the differences we found across the generations may be partly explained by the gender imbalance. Furthermore, we deliberately sampled participants diagnosed over a period that spanned the breadth of HIV history, but the inclusion of participants from different HIV generations also meant that they had different recall periods and were at different stages of their HIV journey. As with all interview‐based research, there is also a risk of social desirability bias and single interviews can only provide insight into what people say they do rather than what they actually do.^29^

### 4.2 Implications

People with HIV today live with more possibility and promise than ever before, with minimal disruption to their lives if diagnosed early and the freedom to pursue careers, relationships and lifestyles similar to the non‐HIV population. The decline of mutual support through activism set against a background of shrinking ancillary HIV support services, in part due to the improved treatment of HIV, has paradoxically increased the dependency of people on clinical services to provide wider social support in addition to monitoring and safeguarding their physical health and prescribing their medication. HIV clinical outcomes in the UK have been good; the care continuum currently exceeds UNAIDS targets in terms of starting treatment, retention and viral suppression of those in care.^30^ However, this biomedical transformation has not yet translated to corresponding improvements in the social construction of HIV in wider society—which must be overcome to enable taking the final step towards the normalization of HIV as an unexceptional chronic illness. Public education campaigns that would bring the wider population up to date with developments in the HIV world are urgently required. To continue to deliver “person‐centred care”^31^ in the UK, we must keep sight of the fact that HIV remains a complex condition that continues to be stigmatized and therefore specialized clinical and support services are still necessary. It is crucial that we build on the resilience of people with HIV to seek individual and collective ways of maintaining and improving services that are essential to living well with HIV.

## CONFLICT OF INTEREST

All the authors of this manuscript declare they have no conflict of interest.
