# Supplementary material for: From activism to secrecy: Contemporary experiences of living with HIV in London in people diagnosed from 1986 to 2014
Source: Health Expect. 2018 Aug 30;21(6):1134–41. doi: 10.1111/hex.12816 (PMC6250870; doi:10.1111/hex.12816)
Supplement: Supplementary file 1 [file HEX-21-1134-s001.docx]

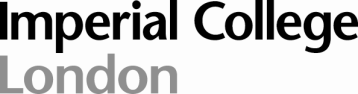

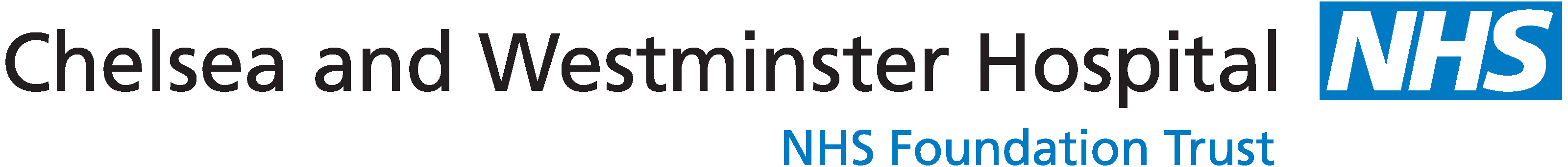


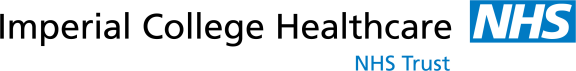


**Appendix S1.**

**HIV patient interview topic guide**

**Overview description (recap and summary of information in the patient information sheet)**

During this project we wish to find out people’s own experiences of their life with HIV starting from the beginning, before you were diagnosed and going all the way up to the present. There are some specific areas we wish to cover, however you are free to discuss what you feel are important issues or areas that have been relevant in your HIV journey.

**Questions:**

*[May or may not need to ask all the questions specifically, this is just a guide, some questions overlap]*

1. **Personal information – work/relationships/home situation**

Can you tell me a bit about yourself….

- 1. What are your home circumstances?
  2. Are you in a relationship/s?
  3. Do you have dependents children? Parents? partner ?
  4. Do you work? What do you do? Benefits?

1. **Testing decisions**
   1. Can you tell me when you were diagnosed?
   2. How did you come to be tested?
   3. Where did you go for your test? Why?
   4. Had you had HIV tests before, if so why?
   5. What was your health like before your diagnosis?
   6. Had/did you know anybody living with HIV at the time?
2. **Experience of initial care**
   1. What happened when you were given your positive result?
   2. Can you remember what information you were given at the time? Was this given in the way you wanted? Please can you tell me in detail.
   3. What questions, if any, did you have for the clinical staff? Did you ask them?
   4. Did you tell anyone / talk to anyone after you found out your results? Can you tell me a bit more about this – who were they, was it helpful to talk to them, etc.?
   5. Did you access information about HIV? How? where? How has this helped?
   6. What did you do after receiving your diagnosis?
   7. Which HIV clinic did you go to? Why? Are you still at that clinic? Why?
3. **Treatment decisions and research**
   1. Are you on treatment ?
   2. How was the decision to start taken? Were you involved in that decision?
   3. What preparation did you make?
   4. (If not on treatment) What are your thoughts about going on treatment?

(If on treatment) What do you think / have you any thoughts about being on treatment?

- 1. (If on treatment) How do you find taking your medication?
  2. Have you discussed your thoughts about treatment with anyone?
  3. Do you know what recommendations there are for when people might start treatment?
  4. Are you aware of the research into ARVs and treatment guidelines in the UK?
  5. What do you know about them? What do you think of them?
  6. How have they influenced your understanding of treatment?
  7. Have they influenced your decision to start/change/stop treatment?
  8. Do you take part in clinical trials? Why?

1. **Clinical care**
   1. How often do you go to the clinic? Has that changed over the time you have been in the clinic? How?
   2. Who do you see? And for what? Doctor? Consultant? Nurse? Peer support? Health Advisor? etc
   3. What kind of care have you received at the clinic? Any good/bad experiences?
   4. What do you think the role of the clinic is in your care?
   5. What other clinical services do you access? Where? How is your care coordinated?
   6. What do you think about the way in which HIV services are currently organised? Does it work for you?
   7. Have they changed? In what way?
   8. Are you aware of possible future changes in the services?
2. **Primary care /Community and Social care**
3. Where do you go for your primary care needs? Why? What is your experience of primary care?
4. How is your care coordinated between primary care and the clinic?
5. Do you access any social care or voluntary services? If so what? Why?
6. Do they meet your needs? If not why?
7. Have you seen any changes in what is available? If so what?
8. **Living with HIV**
   1. Has having HIV affected your life? What are the most significant ways HIV has affected your life, and has that changed over time?
   2. Do you know others living with HIV? What impact does that have on your life?
   3. What kind of support have you had/needed since you were diagnosed positive? Has this changed over time?
9. **Is there anything else you would like to say?**
   1. Given your experience of living with HIV, what would you say to others who have been recently diagnosed about what to expect?
